# Clinicopathologic Profile and Psychosocial Experiences of Nigerian Breast Cancer Survivors

**DOI:** 10.1200/GO.23.00022

**Published:** 2023-09-28

**Authors:** Funmilola Olanike Wuraola, Olalekan Olasehinde, Matteo Di Bernardo, Adewale Abdulwasiu Aderounmu, Adewale Oluseye Adisa, Oluwatosin Zaniab Omoyiola, Adeleye Dorcas Omisore, Thomas Peter Kingham, Victoria Mango, Olusegun Isaac Alatise

**Affiliations:** ^1^ Surgery Department, Obafemi Awolowo University, Ile-Ife, Nigeria; ^2^ Surgery Department, Obafemi Awolowo University Teaching Hospitals Complex, Ile-Ife, Nigeria; ^3^ Massachusetts Institute of Technology, Cambridge, MA; ^4^ Morbid Anatomy and Forensic Medicine Department, Obafemi Awolowo University Teaching Hospitals Complex, Ile-Ife, Nigeria; ^5^ Radiology Department, Obafemi Awolowo University, Ile-Ife, Nigeria; ^6^ Memorial Sloan Kettering Cancer Centre, New York, NY

## Abstract

**PURPOSE:**

Breast cancer survivors are a distinct category of patients with unique characteristics and needs. The population of survivors is expected to increase, given the rising incidence of breast cancer in Nigeria, and the improvements in breast cancer outcomes. This study evaluated the clinicopathologic characteristics and the psychosocial experiences of a cohort of Nigerian breast cancer survivors.

**METHODS:**

From an institutional breast cancer database, patients managed between January 2010 and December 2016 were evaluated. Clinicopathologic characteristics, treatment details, and survival estimates were assessed. These were compared with nonsurvivors managed during the same period. Survivors were defined as those who have been alive for at least 5 years from the date of presentation. Qualitatively, a purposive sample of 20 survivors was evaluated using one-on-one in-depth interviews to assess their experiences and coping mechanisms after treatment.

**RESULTS:**

Of the 355 patients in the database during the study period, there were 163 survivors (45.9%), while 192 (54.1%) died. Age, stage at presentation, tumor size, and receipt of multiple treatment modalities were significantly associated with survival. Five themes were identified in qualitative analysis: initial reaction to the diagnosis, experiences during treatment, social support, coping strategies, and advocacy. Strong family support and spirituality were prominent coping strategies identified in this cohort.

**CONCLUSION:**

Despite obvious infrastructural and manpower limitations, Nigerian patients who present early and receive multimodal therapy and different breast cancer treatments have better odds of survival. Survivors have some unmet psychosocial and physical needs requiring intervention.

## Teaser

Breast cancer survivors mitigated the impact of the diagnosis with physical enhancement, spirituality, and family support.

## INTRODUCTION

Breast cancer is the most common female malignancy worldwide; although the incidence is highest in high-income countries (HICs), breast cancer mortality is disproportionately higher in low- and middle-income countries (LMICs).^1-3^ Over the past few decades, LMICs have continued to experience a significant increase in breast cancer incidence with generally poor outcomes. In Nigeria, breast cancer incidence is about 54.3 per 100,000 population, which is approximately a 100% increase within the past decade.^1^ Moreover, it is currently one of the leading causes of cancer deaths in Nigeria.^4^

**CONTEXT**

**Key Objective**
To identify the clinicopathology differences in breast cancer survivors versus nonsurvivors and the impact of the diagnosis on survivors.
**Knowledge Generated**
This study shows that small tumor size, short intervals of presentation, early cancer stage, and multimodal treatments are associated with improved survival. The impacts of breast cancer on the participants were mitigated using different coping strategies, which range from physical enhancement with the locally fabricated prosthesis to spirituality and family support.
**Relevance**
From this study, it's evident that improving the public awareness about breast cancer, early presentation, and provision of multimodal treatment will help improve the outcome of breast cancer in Nigeria. Having a sustainable policy that will improve early detection and treatment will have a significant impact on outcomes. Setting up a survivor program in Nigeria should include spirituality and family support as an essential component.

In Nigeria, the majority of patients with breast cancer present in advanced stages.^5^ Poor knowledge about breast cancer, sociocultural beliefs, lack of access to screening and diagnostic facilities, primary care delay, and poverty are some of the factors contributing to this trend.^6,7^ A recent review reported an overall 5-year breast cancer survival rate in Nigeria of 43%,^5^ which is far less than the 90% 5-year estimates reported in many HICs. Although outcomes are generally poor, there have been some marginal improvements over the years. This might be related to improvement in disease presentation and access to diagnostic and treatment facilities witnessed in recent years.^5^

As outcomes improve, issues relating to breast cancer survivorship require rigorous investigation. In HICs, survivorship is a significant component of cancer care. Breast cancer survivors tend to face various physical, psychosocial, and emotional challenges. They have been shown to have the highest disability-adjusted life-years compared with other malignancies.^8^ In LMICs, where most efforts are geared toward achieving an early diagnosis and access to treatment, very little attention is paid to survivorship. Understanding the clinicopathologic characteristics and the experiences of survivors might help identify areas requiring interventions aimed at improving patients' overall outcomes. In Nigeria, very limited data exist on this subject. The purpose of this study is to compare the clinicopathologic characteristics of Nigerian breast cancer survivors with nonsurvivors and qualitatively explore survivors' experiences with breast cancer and breast cancer treatment.

## METHODS

This prospective study was carried out in the General Surgery Units of the Obafemi Awolowo University Teaching Hospitals Complex (OAUTHC), Ile-Ife, Osun State, Nigeria, with institutional review board (IRB) approval and compliance with patient privacy policies (IRB Protocol number ERC/2022/01/21), using the African Research Group on Oncology's breast cancer database, which is a prospectively maintained database and biorepository.

The study population comprised patients age 18 years and older, males and females, diagnosed with breast cancer between 2010 and 2016 who were captured in the database. Twenty patients enrolled in the qualitative part of the study were based on data saturation.

### Study Protocol

#### Study Design

This is a mixed-method study. Survivors were defined as those who have lived for at least 5 years from diagnosis, while nonsurvivors were defined as those who had died within 5 years of diagnosis.

#### Quantitative Analysis

Data were accumulated from a REDCap database of breast cancer cases of patients presenting at OAUTHC. To ensure that cases had a minimum of 5 years of follow-up, only cases that presented between January 1, 2010, and December 31, 2016, were analyzed. The total number of cases captured during this period and entered into the database was 374, but only 355 cases with known follow-up status were included in the analysis (5.1% lost to follow-up). Specific variables pulled from the database were age (years), sex, duration of symptoms, mass size (cm), overall clinical stage (at presentation), histology, Nottingham grade, immunohistochemistry, and treatment received. The data were largely complete for all variables analyzed. The comparison was made between the survivors (5 years and above from diagnosis) and nonsurvivors.

#### Qualitative Analysis

From the database, survivors were contacted purposively by telephone, taking into consideration their age group to ensure fair representation across different age groups (below 45 years at diagnosis, between 45 and 64 years, and above 65 years at diagnosis). Those who verbally consented after the invitation were invited to the outpatient clinic where written consents were obtained. They had a one-on-one in-person in-depth interview to explore their quality of life (QOL), coping mechanisms, and the effects of breast cancer on their lives. Interviews were conducted by a trained qualitative researcher (the research assistant) in a private environment in the hospital. Debriefing and member checking was done to ensure reliability. All interviews were conducted in the language patients were most comfortable with (English and/or Yoruba), and transcribed for analysis.

### Statistical Analysis

#### Quantitative Analysis

All statistical analyses were done in R-studio 1.3.1073, using R v4.0.2 (R Foundation for Statistical Computing, Vienna, Austria; R-Studio: Posit Software, PBC, Boston, MA). Various sociodemographic and clinical variables were used to provide descriptive statistics of survivors (163) and nonsurvivors (192; Data Supplement, Appendix 1). Various statistical comparisons were performed to analyze different variables, including the Welch two-sample t-test for age, complaint duration and mass size, the Wilcoxon rank-sum test for age (grouped), complaint duration (grouped), overall clinical stage, Nottingham grade and the 2- proportion z-test for sex, overall clinical stage (grouped), immunohistochemistry, and treatment options. Univariate Cox proportional hazards regression models were run using the Coxph package to assess the impact of each clinical variable on survival, with age and complaint duration variables grouped into appropriate categorical groups. To assess the proportional hazards assumption, we examined Schoenfeld residuals for each variable in the Cox model. Multivariate analysis was also conducted to examine the association between the clinical variables and survival outcomes. Survival analysis was done using a log-rank test (*survminer* package) for grouped overall clinical stages, and Kaplan-Meier survival curves were generated and presented for this specific comparison.

#### Qualitative Analysis

The patients' interviews were translated when necessary, then transcribed and coded to identify quotes, categories, and themes that address the stated objectives using the Atlas Ti software version 8 (Scientific Software Development GmbH, Berlin, Germany). The analytical framework and codes for the qualitative analysis were developed. Transcripts were analyzed, and recurrent, dominant, and divergent narratives were identified. Quotes bearing meaningful concepts were identified, categorized, and labeled.

## RESULTS

### Quantitative Analysis

**FIG 1:**
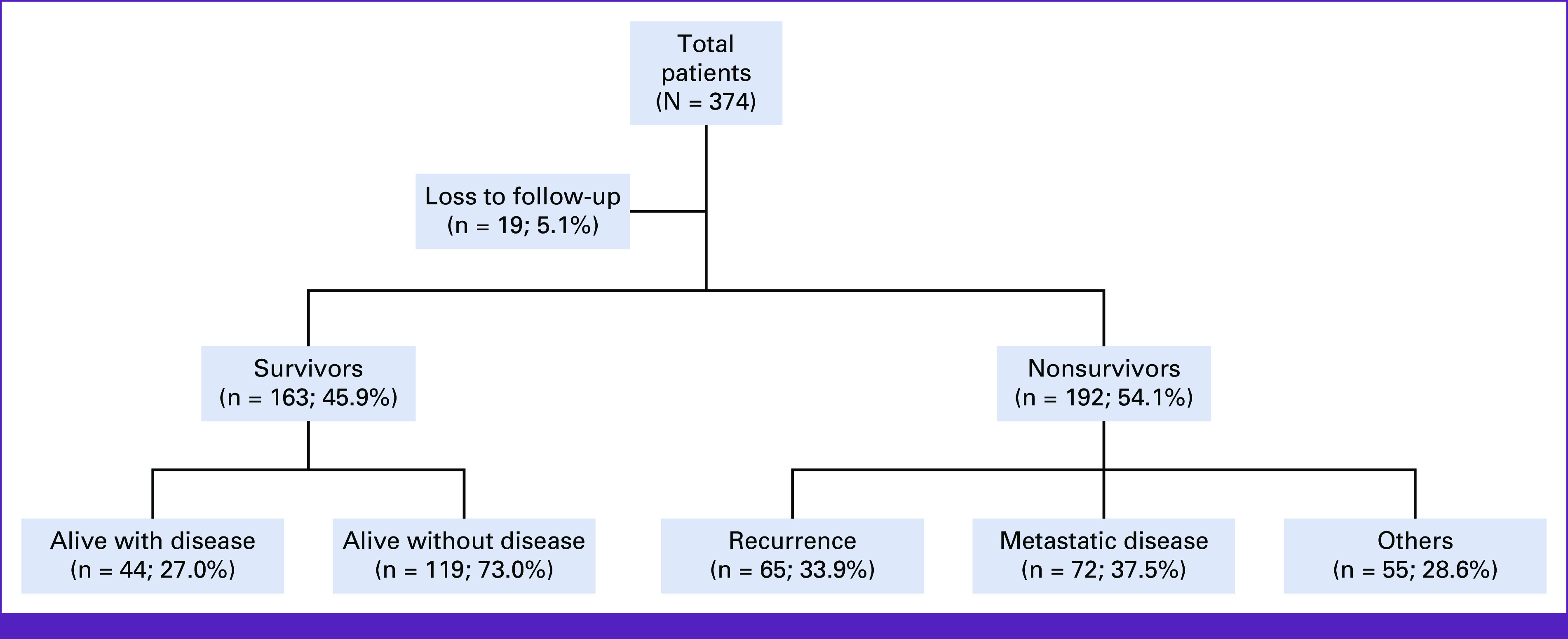
Flowchart describing the patients in the breast cancer database.

A total of 355 patients were analyzed, 192 were nonsurvivors (54.1%) and 163 survivors (45.9; Fig 1). Of the 355 patients, 347 (97.7%) are females and 8 (2.3%) males, with a median age of 49.8 years. Forty six (13%) were stage I/II, while 309 (87%) were stage III/IV. Chemotherapy use: neoadjuvant 281 (79.2%), adjuvant 156 (43.9%), and palliative 47 (13.2%). Hormonal treatment in 94 (26.5%), Herceptin in 10 (2.8%), and surgery in 224 (63.1%).

#### Clinicopathologic Comparison of Survivors and Nonsurvivors

The mean age (at presentation) of survivors was 50 years, while that of nonsurvivors was 48 years (*P* = .02). Of the survivors, 25 (15%) were young (39 years and below), and 52 (27%) were nonsurvivors (*P* = .01; Table 1). Overall, few patients had immunohistochemistry analysis conducted, but this was higher among survivors (n = 39, 24%) compared to 23 patients (12%) among nonsurvivors (*P* =.004). Biomarker status (estrogen receptor, progesterone receptor, human epidermal growth factor receptor 2, triple-negative) was not significantly different between the two groups. Treatment, such as adjuvant chemotherapy, (56% of survivors, 34% of nonsurvivors, *P* =.0005), surgery (77% of survivors, 51% of nonsurvivors, *P* =.00003) and radiotherapy (17% of survivors, 8% of nonsurvivors, *P* =.04) were higher in survivors than nonsurvivors. Finally, multimodal therapy (a combination of surgery, chemotherapy and radiotherapy) was carried out in 27 (17%) survivors and 14 (7%) nonsurvivors (*P* =.03).

**TABLE 1:**
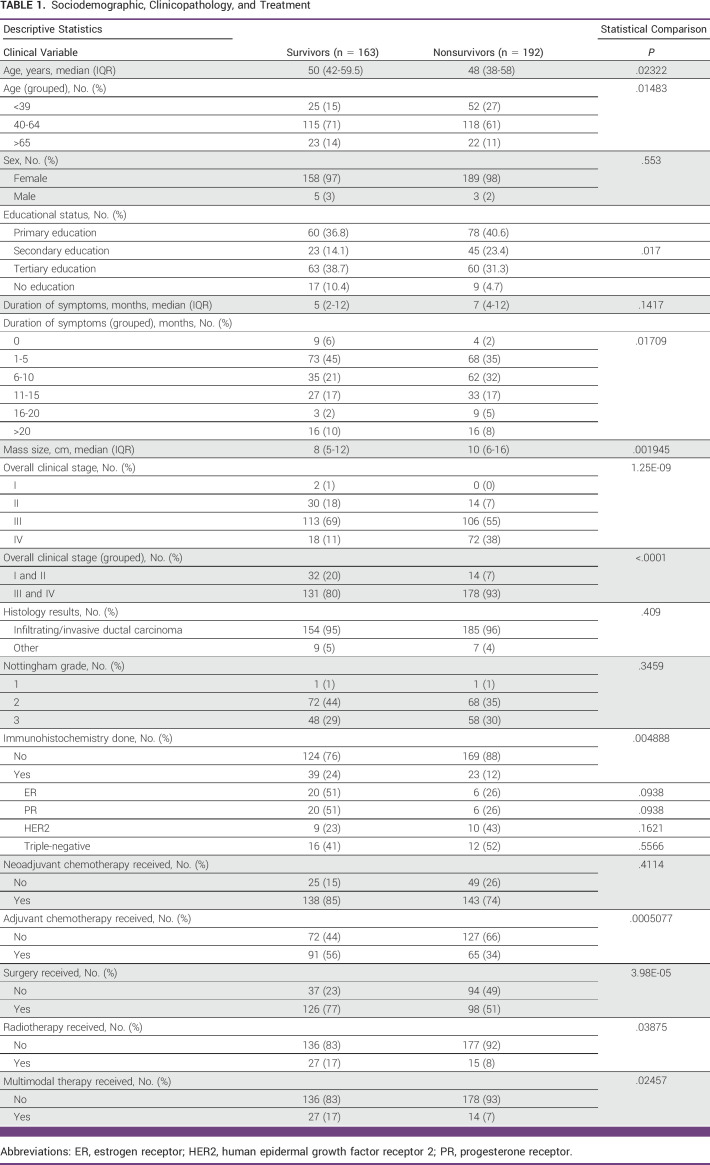
Sociodemographic, Clinicopathology, and Treatment

In nonsurvivors, 72 (38%) were stage IV, while 93% were stage III and IV, while in survivors, only 18 (11%) were stage IV, and 80% were stage III and IV (*P* = 1E-09; *P* = .001).

#### Survival Analysis

**FIG 2:**
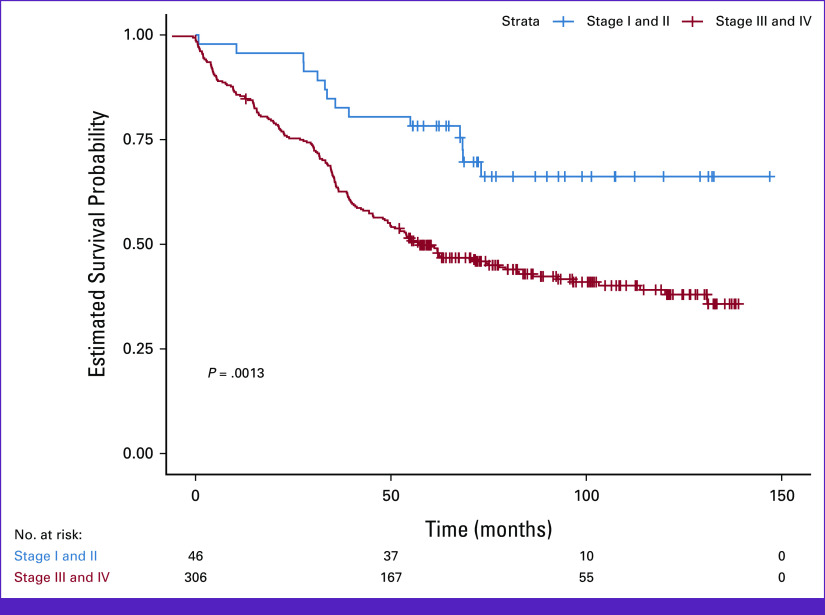
Five-year survival for patients stratified by early (stage I and II) and late (stage III and IV) overall stage.

Positive and significant coefficients found (hazard ratio [HR]; 95% CI presented in parentheses) were mass size (HR, 1 [1 to 1.1]; *P* = .000096), overall clinical stage (HR, 2.6 [2 to 3.4]; *P* = 2E-13), and grouped overall clinical stage (HR, 2.4 [1.4 to 4.1]; *P* = .0018; with increasing values of these variables, a significantly higher risk of death was found; Table 2). Negative and significant coefficients included grouped age (HR, 0.72 [0.56 to 0.93]; *P* = .01) and multimodal therapy (HR, 0.45 [0.26 to 0.78]; *P* = .004; with increasing values of these variables, a significantly lower risk of death was found; Table 2). Overall survival (OS) for stages I and II compared with III and IV was statistically significant (log-rank *P* = .0013; Fig 2). Median survival for groups with an event is shown in the Data Supplement (Appendix I).

**TABLE 2:**
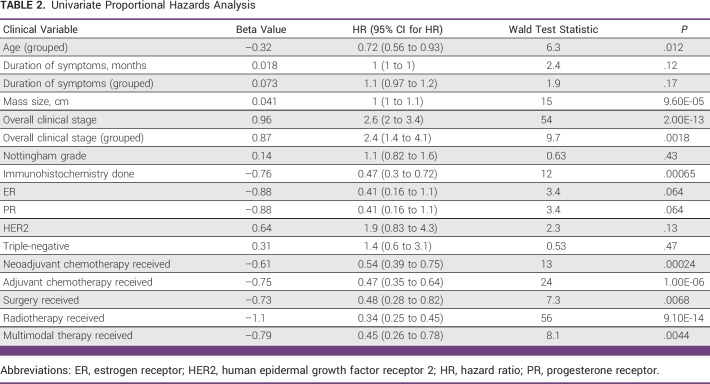
Univariate Proportional Hazards Analysis

### Qualitative Analysis

The age of respondents ranged from 38 to 68 years, and all were married (Table 3).

**TABLE 3:**
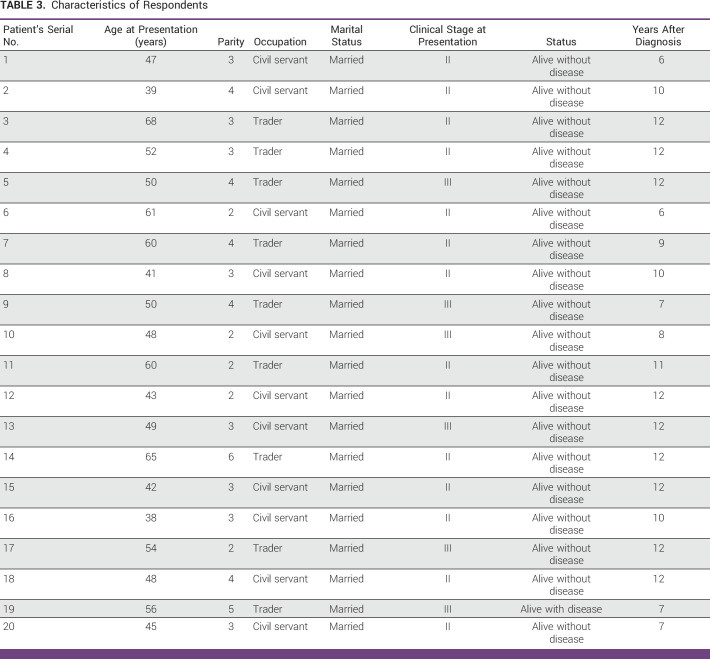
Characteristics of Respondents

Six themes were identified (Data Supplement, Appendix I).

#### Initial Reaction to the Diagnosis

Participants had different reactions to the diagnosis of breast cancer, which they expressed in a variety of ways, such as denial, surprise, and sadness. A 60-year-old respondent described her reaction of both surprise and sadness: “*I was like me? Cancer? I cried a lot because I was 60 years then so I was wondering at that age?*”

#### Disclosure

The result of the diagnosis was difficult to share with people. Nevertheless, most of the breast cancer survivors informed their immediate family members, mostly their spouses and their children. The rationale for telling them was to seek support and help in decision making relating to the treatment plan. A 38-year-old respondent said: “*My husband because that's the closest person to me*.”

#### Experiences During Treatment

The respondents had various experiences from diagnosis to completion of treatment. The side effects of chemotherapy were considered by many as a challenge as it changed their body systems, which made it obvious that they were ill. A 65-year-old respondent said: “*The chemotherapy is the most challenging because it changed my body and my hair, always vomiting and weak*.”

#### Social Support

Individuals whom the survivors informed about the diagnosis were helpful to them. The person assisted in several ways such as financial, providing support during hospital visits, helping with child care, decision making, and general support. A 41-year-old respondent highlighted how her spouse encouraged her to receive treatment: “*Yes, going by the doctor's advice, he also opted for it and we both decided on it*.”

#### Coping Strategies With the Effect of Diagnosis and Treatment

Survivors used a variety of coping techniques, which include physical, psychological, and spiritual coping methods. An interesting social coping strategy that was noted among participants in this study was concealment of their health conditions as a way of avoiding stigmatization: “*No, you know something like that, if everyone knows what you are passing through, they will be discriminating. I had my treatment and went for radiotherapy and to the glory of God, they did not know anything*.”

#### QOL of Breast Cancer Survivors

Breast cancer survivors' assessments of their QOL were based on the vistas of possibilities that they envision for themselves, and are thus an important part of their identity. The data identified three domains of QOL among respondents, which are social life, family life, and sexual life.

##### The social life of survivors.

Although many attended social functions, they alluded to the limitations they experienced in terms of their appearance. A 43-year-old and 42-year-old, respectively, said: “*No, just that I can't wear the styles of clothing the way I like but I go out without no issue within me or from anyone*.”

And “*No, it didn't have any effects just that I can't remove my clothes anywhere or anyhow unlike before if you go for programs with your friends you might want to bath together, I don't do that again because they were not aware of it*.”

##### Family life of survivors.

Family life refers to the type of life a survivor lived when they are married and have children, as well as how their condition affects or causes numerous issues among spouses, children, and relatives. A 52-year-old respondent describes the role of her husband.

“*My husband was there for me despite all I went through, it didn’t affect my going out or anything in relating with anyone or my children*.” Another respondent also narrated the role her children played: “*No, it didn’t affect any relationship then my husband was late and my children were there for me*.”

A respondent expressed how her relatives rallied around her: “*No, it didn't affect anything because its only my immediate family that's aware of what I went through, so no one knows anything about it, and I don't have any issue with my spouse and my children*.”

##### Sexual life of survivors.

The effect of breast cancer treatment on respondents varied from one patient to another. These respondents age 61 years and 45 years reported, respectively: “*(smiles) It doesn't have any negative effect on my sexual life besides I'm not a young lady anymore so there's no pressure or issue about sex and having children, my children were even grown-ups then*.”

“*No, I’m of age because by that time I was around 45 and we’ve decided we are not having more kids*.”

A younger patient said it affected her sex life during treatment but her spouse was understanding: “*I don't feel like having sex my husband always encourages me and eventually I can get to have sex again*.”

A 39-year-old said: “*Yes, it affected my sex life and reduces my chances of having more children*.”

#### Advocacy

Breast cancer survivors' advice or suggestion was that women should constantly observe self-breast examinations and always go to the hospital for medical checks.

A 52-year-old woman: “*My advice is that women should check their breasts and if any observation they should visit the hospital and yield to the doctor's advice*.”

A 47-year-old woman: “*Doctors should provide breast reconstruction*.”

## DISCUSSION

This study identified some favorable clinicopathologic factors among breast cancer survivors in this Nigerian cohort. Longer-term survival was associated with patients with smaller masses, who were older, with shorter symptom duration, lower clinical stage, full pathology workup (immunohistochemistry), and the receipt of multimodal therapy. This study also identified some of the negative experiences of patients after treatment and highlights some key coping strategies such as social/family support, spirituality, and avoidance of stigma using various approaches.

Age at diagnosis is associated with survival, the median age of patients in this cohort was only slightly different, and there was a significantly lower proportion of younger women among the survivors relative to nonsurvivors (15% *v* 27%). Similar findings have been reported in other studies.^9-11^

Early detection has been associated with improved survival. Studies have shown that mammographic screening reduced breast cancer–related mortality by about 10%-25%.^12-14^ The OS for metastatic disease is about 27%-30% compared with regional disease which is about 86%.^5,15,16^ In this study, both duration of symptom and stage of presentation were lower in survivors than nonsurvivors.

In this cohort, none of the patients had screen-detected lesions because of Nigeria's lack of an organized screening program. Survivors were, however, found to have smaller masses than nonsurvivors. Previous studies also show a direct relationship between tumor size and outcomes, with large tumor size and nodal involvement being associated with poor outcomes.^17-19^ Although it may be desirable to eventually have an organized mammography screening program, our findings suggest that investing in programs that promote early diagnosis will be a more pragmatic and less expensive way of improving breast cancer survival in Nigeria. Adopting the WHO Global Breast Cancer Initiative pillars should be encouraged.^20^

Tumor grade has been associated with survival in some studies.^21-23^ Rakha et al^22^ found that the higher the tumor grade, the worse the prognosis, although the same study also concluded that the presence of positive lymph node also affects outcome irrespective of the grade. In this study, the grade was not associated with survival. Generally, the majority of cases in both cohorts were high grade, suggesting that breast cancer in our study population may be biologically aggressive.

The majority of patients in this cohort presented with locally advanced disease; treatment guidelines stipulate the addition of radiation therapy in addition to surgery and chemotherapy. Radiotherapy is quite challenging to access in Nigeria.^24^ This fact is reflected in this study, with only 12% of patients receiving radiotherapy. This study also found that receipt of multimodal treatment (11.5% of patients in the overall cohort) was associated with better survival. This aligns with other studies that have demonstrated the significance of multimodal therapy.^25-28^ Improving access to care by creating more radiotherapy facilities and subsidizing treatment through health insurance schemes is an area of intervention that might result in huge gains in terms of survivorship.

The participants mitigated the impact of breast cancer diagnosis in different ways. Patients resorted to various coping strategies to augment their physical appearance after mastectomy. Body image is a significant factor in breast cancer care that merits due consideration.^29^ Women in this study use locally made prostheses over the mastectomy site. The development of breast reconstruction services and the promotion of breast-conserving operations in qualifying patients will significantly help patients cope better with the body image challenges associated with breast cancer treatment.

Generally, patients appeared to be quite conservative regarding sexual issues. This is quite typical of African women who rarely discuss sexual issues because of cultural and religious biases. In the African context, discussions relating to sexual issues are often regarded as taboo.^30-32^ Although many of the women reported minimal impact on their sexual life, the effect of breast cancer treatment on sexual and family life, in general, was mostly felt by younger patients whose desire for additional children was disrupted. It is also noteworthy that increased sexual intimacy was identified by a participant as a way by which emotional support was provided during treatment.

The role of spirituality as a coping mechanism is clearly highlighted in this study. Although spirituality has also been identified as a coping strategy in other parts of the world,^33-36^ religion is known to have a huge impact on the lives of people in Nigeria and in Africa in general. The inclusion and engagement of religious leaders as advocates can improve the early presentation and compliance with treatment. There is an association between stigma and late presentation, acceptance of treatment, and negative impact on the QOL of survivors. Patients with good family support have been shown to have better outcomes.^37,38^ There is a need for increased public awareness about breast cancer to reduce stigmatization and its consequences on outcomes.

The fewer number of relatively younger patients interviewed might have affected the responses obtained particularly in certain domains such as sexuality and social interactions, and this can affect the generalization of the result. This was mitigated by selecting patients for qualitative interviews on the basis of age.

Future plans include promotion of early detection, breast reconstruction, and breast-conserving operations as well as awareness to break the barrier of stigma and promote better social networking among breast cancer survivors.

In conclusion, this study identifies important clinical and pathologic characteristics associated with survival, some of which are modifiable and can be included in public health interventions. Such measures include interventions that will shorten the diagnostic interval of patients with breast cancer, promote access to effective therapies, and improve the outcome of breast cancer survivors.
